# The Impact of COVID-19 on Conspiracy Hypotheses and Risk Perception in Italy: Infodemiological Survey Study Using Google Trends

**DOI:** 10.2196/29929

**Published:** 2021-08-06

**Authors:** Alessandro Rovetta

**Affiliations:** 1 Mensana srls Brescia Italy

**Keywords:** COVID-19, fake news, Google Trends, infodemiology, Italy, risk perception

## Abstract

**Background:**

COVID-19 has caused the worst international crisis since World War II. Italy was one of the countries most affected by both the pandemic and the related infodemic. The success of anti–COVID-19 strategies and future public health policies in Italy cannot separate itself from the containment of fake news and the divulgation of correct information.

**Objective:**

The aim of this paper was to analyze the impact of COVID-19 on web interest in conspiracy hypotheses and risk perception of Italian web users.

**Methods:**

Google Trends was used to monitor users’ web interest in specific topics, such as conspiracy hypotheses, vaccine side effects, and pollution and climate change. The keywords adopted to represent these topics were mined from Bufale.net—an Italian website specializing in detecting online hoaxes—and Google Trends suggestions (ie, related topics and related queries). Relative search volumes (RSVs) of the time-lapse periods of 2016-2020 (pre–COVID-19) and 2020-2021 (post–COVID-19) were compared through percentage difference (∆_%_) and the Welch *t* test (*t*). When data series were not stationary, other ad hoc criteria were used. The trend slopes were assessed through Sen slope (SS). The significance thresholds have been indicatively set at *P*=.05 and *t*=1.9.

**Results:**

The COVID-19 pandemic drastically increased Italian netizens’ interest in conspiracies (∆_%_ ∈ [60, 288], *t* ∈ [6, 12]). Web interest in conspiracy-related queries across Italian regions increased and became more homogeneous compared to the pre–COVID-19 period (average RSV=80±2.8, *t*_min_=1.8, ∆_min%_=+12.4, *min*∆_SD%_=–25.8). In addition, a growing trend in web interest in the infodemic YouTube channel ByoBlu has been highlighted. Web interest in hoaxes has increased more than interest in antihoax services (*t*_1_=11.3 vs *t*_2_=4.5; Δ_1%_=+157.6 vs Δ_2%_=+84.7). Equivalently, web interest in vaccine side effects exceeded interest in pollution and climate change (SS_vaccines_=0.22, *P*<.001 vs SS_pollution_=0.05, *P*<.001; ∆_%_=+296.4). To date, a significant amount of fake news related to COVID-19 vaccines, unproven remedies, and origin has continued to circulate. In particular, the creation of SARS-CoV-2 in a Chinese laboratory constituted about 0.04% of the entire web interest in the pandemic.

**Conclusions:**

COVID-19 has given a significant boost to web interest in conspiracy hypotheses and has made it more uniform across regions in Italy. The pandemic accelerated an already-growing trend in users’ interest toward some fake news sources, including the 500,000-subscriber YouTube channel ByoBlu, which was removed from the platform by YouTube for disinformation in March 2021. The risk perception related to COVID-19 vaccines has been so distorted that vaccine side effect–related queries outweighed those relating to pollution and climate change, which are much more urgent issues. Moreover, a large amount of fake news has circulated about COVID-19 vaccines, remedies, and origin. Based on these findings, it is recommended that the Italian authorities implement more effective infoveillance systems, and that communication by the mass media be less sensationalistic and more consistent with the available scientific evidence. In this context, Google Trends can be used to monitor users’ response to specific infodemiological countermeasures. Further research is needed to understand the psychological mechanisms that regulate risk perception.

## Introduction

COVID-19 was responsible for one of the most dramatic global crises after World War II. As of April 24, 2021, the official global toll was 144 million cases and 3.1 million deaths [1]. Such a pandemic has also triggered a vast infodemic, capable of seriously damaging the economic and health systems of many countries as well as enabling the spread of the novel coronavirus itself [2]. Specifically, an infodemic is defined as an excessive amount of unfiltered information concerning a problem, such that the solution is made more difficult [3]. However, it is not the first time that the world has been forced to face a vast infodemic; for example, during the HCoV-EMC/2012 (human coronavirus–Erasmus Medical Center/2012) epidemic generated by a previous coronavirus, some flawed denominations, such as “Middle East respiratory syndrome” and “swine flu,” have caused unintentional adverse social and economic impacts by stigmatizing industries and communities [4]. In addition, the adoption of improper names has also led to medical and nursing errors concerning drug administration [5]. To deal with this growing problem, fomented by increasingly rapid mass media such as that provided by the internet, Dr Gunther Eysenbach has devised a scientific branch called “infodemiology,” which encompasses all the techniques for monitoring and analyzing information [3]. In general, the infodemiological approach is based on the collection of information circulating in a network—not necessarily online—with the following possible purposes: (1) investigate the mental and physical health of a group or community, (2) identify the dangers and extent of disinformation or misinformation regarding a specific topic, and (3) carry out assessments in the field of public health (eg, using web searches to obtain information about symptoms and spread of a disease).

As reported by the World Health Organization (WHO), the COVID-19 infodemic can intensify or lengthen outbreaks. For this reason, a huge infodemiological effort has been made to study the information circulating on the web and contain the spread of fake news [6]. In this context, 132 nations worldwide signed a document to guarantee their commitment to the battle against disinformation and misinformation [7]. On the operational level, infodemic management takes place through four key steps: (1) listening to community concerns and questions, (2) promoting understanding of risk and health expert advice, (3) building resilience to misinformation, and (4) engaging and empowering communities to take positive action [2]. This paper focuses on points 2 and 3 as concerns Italy, one of the nations most affected by COVID-19 [1]. The objective is to analyze and quantify the impact of COVID-19 on Italian netizens’ risk perception and new and pre-existing conspiracy hypotheses through Google Trends, an infoveillance tool provided by Google that returns users’ web interest in specific topics in the form of normalized values called relative search volumes (RSVs) [8]. In this regard, pre-existing conspiracies are defined as those conspiracies that existed even before COVID-19 and are not directly related to it. The denomination “conspiracy hypotheses” aims to underline the absence of the scientific background necessary to call them theories. Google Trends has been exploited extensively in the scientific community to conduct infodemiological, medical, psychological, economic, and even epidemiological studies [9-14]. Indeed, although the media can influence users’ web searches [15], Google Trends provides valuable details on the dynamics of users’ online interests, including the influence of the media on collective thinking [16,17].

As of April 2021, the success of the vaccination campaign has been crucial in the fight against COVID-19 [18,19]. Conspiracy hypotheses, inadequate risk perception, and unjustified fears have already undermined nonpharmacological containment measures and can reduce the effectiveness of pharmacological ones [20]. Furthermore, these factors can compromise the management of equally serious problems such as pollution and climate change, which are often linked to COVID-19 incidence and mortality [21-23]. Therefore, such a scenario requires careful surveillance of the online information flow as well as thoughtful communication. As we will show in this research, Google Trends can help achieve this goal.

## Methods

### Data Collection

#### Overview

For each topic, appropriate keywords were selected according to the methods explained in the following subsections. Each keyword was searched on Google Trends under the category “all categories.” The time-lapse period was set to 5 years (April 21, 2016, to April 21, 2021). Only the most relevant queries were included in the results (ie, 
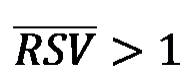
). The selection of the queries with the highest RSVs was conducted by consulting related topics and related queries provided by Google Trends. In this way, it was possible to select the most relevant queries, including those containing typos. All keywords were collected for at least 7 consecutive days in order to highlight potential anomalies and significant variations [24].

#### Pre-existing Fake News

Pre-existing fake news and disinformation channels were mined from the specialized antihoax website Bufale.net [25]. The selection of the keywords to search on Google Trends took place through the following steps: (1) consultation of the blacklisted infodemic sources drawn up by the authors of the Bufale.net website, (2) search of all the aforementioned infodemic sources on Google Trends, and (3) selection of infodemic sources with 
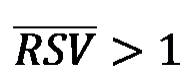
. By doing so, four keywords that represent the main conspiracy-related web interests on Google Trends were identified: “cospirazione + nuovo ordine mondiale + complotto” (conspiracy + new world order + plot), “byoblu” (a 500,000-subscriber YouTube channel removed in March 2021 for disinformation), “Maurizio Blondet” (an Italian journalist who supports conspiracy hypotheses), and “luogocomune” (a Facebook page sharing conspiracy hypotheses). All of these keywords have been independently searched on the web to verify the actual presence of hoaxes and fake news, understood as information that conflicts with current scientific literature. The details of this examination are reported in Multimedia Appendix 1.

#### Risk Perception

RSVs of the query “fake news + bufale + notizie false” (fake news + hoaxes + false news) and the previous queries (ie, pre-existing fake news) were compared. By doing so, it was possible to observe the impact of the pandemic on web interest in antihoax services and the hoaxes themselves. The same procedure was carried out for the queries “vaccini effetti collaterali + vaccino effetti collaterali” (vaccine side effects + vaccines side effects) and “inquinamento + cambiamento climatico” (pollution + climate change) queries. In this way, it was possible to evaluate web interest in two very distant topics in terms of health risk and incidence [26-28].

#### COVID-19–Related Fake News

To monitor the trend of fake news in Italy after more than a year of the pandemic, we referred to the following: (1) previous studies conducted during both the first and second waves of COVID-19 in Italy [20,29], (2) the Bufale.net website [25], and (3) the official website of the Italian Ministry of Health [30]. The keywords that reached an 
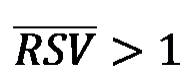
 concerned the following topics: the creation of COVID-19 in a Chinese laboratory (“coronavirus laboratorio + covid laboratorio – analisi – tampone – tamponi”), vaccine plot (“vaccino calamita + vaccino chip + vaccino microchip + vaccino bill gates”), 5G plot (“coronavirus 5g + covid 5g + corona 5g + virus 5g”), COVID-19 plot (“complotto coronavirus + complotto covid + complotto pandemia + grande reset”), and unproven remedies (“coronavirus vitamina + covid vitamina + coronavirus aglio + covid aglio”).

### Statistical Analysis

#### Welch t Test

The Welch *t* test was used independently of the data set distribution, thanks to the central limit theorem (N>30, where N the is the number of measures). Nevertheless, a qualitative graphic control was performed to confirm the absence of too-pronounced skewness. The difference between the two mean values was considered significant indicatively when *t*>1.9.

#### Percentage Change

The percentage change, ∆_%_, was calculated through the formula [*y*(*T*_2_) – *x*(*T*_1_)]/*x*(*T*_1_) × 100, where *T_i_* is a specific time-lapse period.

#### Shapiro-Wilk Test

The Shapiro-Wilk test was used, together with a qualitative graphic control, to evaluate the distributive normality of the data set in question.

#### Mean Values

All mean values were calculated using the standard arithmetic mean and are presented as mean (SEM [standard error of the mean]). When N<30, the Shapiro-Wilk test was performed to assess the goodness of the mean value as a statistical measure.

#### Data Series Analysis

All data series were graphed. To signal the presence or absence of trends, augmented Dickey-Fuller (ADF), Mann-Kendall (MK), and Sen slope (SS) tests were adopted. The same tests were used to evaluate the data sets’ stationarity. Calculations were performed with Microsoft Excel 2021 software through the Real-Statistics 2021 package (Multimedia Appendix 2). The optimal lag was determined using the Schwert criterion.

#### Data Series Comparison

To estimate the effect of COVID-19 on web queries, RSV trends over the last 5 years (April 21, 2016, to April 21, 2021) were analyzed. As shown in a previous paper, Italian netizens showed a marked interest in the COVID-19 pandemic only when it became a direct national problem [31]. Therefore, the time-lapse periods of “April 21, 2016, to February 16, 2020” (period 1) and “February 16, 2020, to April 21, 2021” (period 2) were compared. When period 1 turned out to be stationary or contained a negative trend, *t* and ∆_%_ were calculated. When period 1 contained a stationary positive trend, the trend slopes of period 1 and a specific subperiod of “February 16, 2020, to *x*” of period 2 were compared by ∆_%_; such a subperiod was selected by observing the region of the graph in which a possible positive level-shift occurred. Period 1 data were then linearly, quadratically, or sigmoidally interpolated, depending on which monotone function minimized the statistical errors. Period 2 data were interpolated through a polynomial function of the 9th degree. Finally, ∆_%_ was calculated between the areas subtended by the two curves after February 16, 2020 (∆*A*_%_). These were calculated using a definite integral between weeks 201 and 264. When period 1 contained a positive level-shift but was piecewise quasi-stationary, *t* and ∆_%_ were calculated considering only the last quasi-stationary subperiod.

#### Pearson Correlation

Pearson correlation (*r*) was used only after verifying the distributive normality of the data set through the Shapiro-Wilk test plus a graphical check. No strength thresholds have been adopted. Since all of the samples in which the Pearson correlation was calculated were sufficiently Gaussian, nonparametric correlations were not exploited.

#### *P* Values

Two-tailed *P* values were used as graded measures of the strength of evidence against the null hypothesis. An indicative threshold has been set at *P*=.05; however, exact *P* values for the ADF and MK+SS tests are reported in Multimedia Appendix 2. The remaining *P* values are reported in full in this manuscript.

## Results

### Web Interest in Pre-existing Fake News

**Figure 1:**
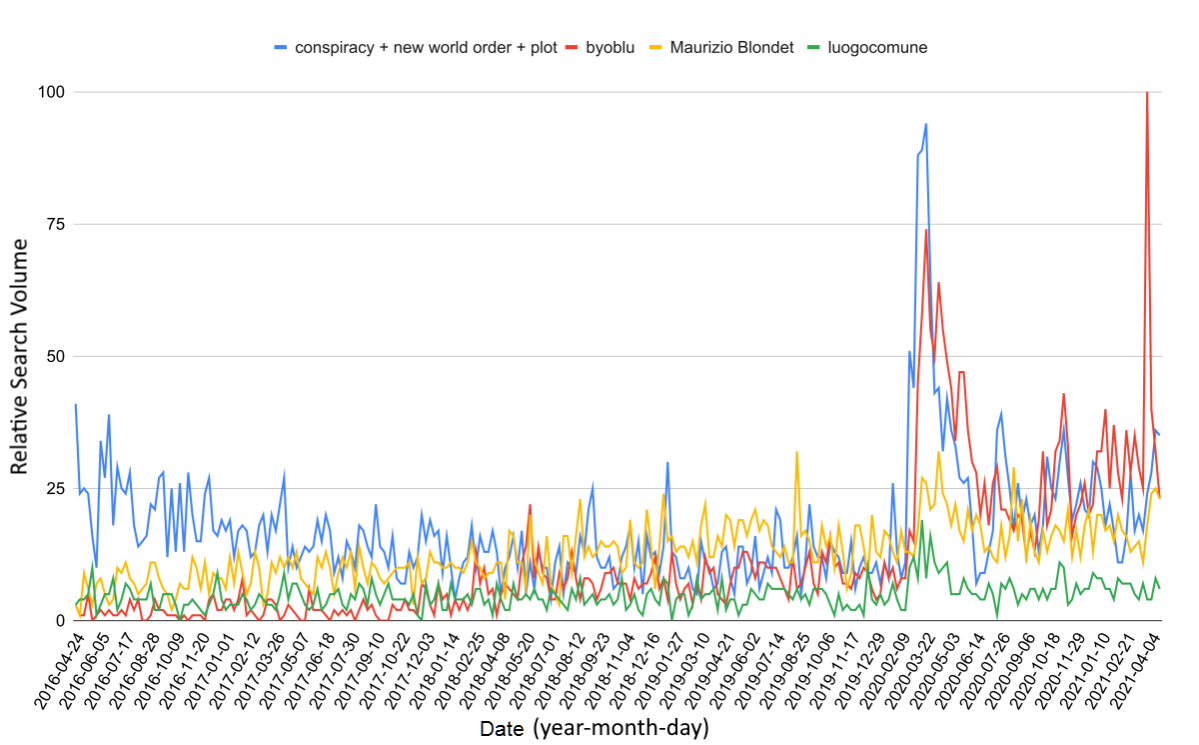
Relative search volumes of conspiracy-related web search queries from April 21, 2016, to April 21, 2021, in Italy.

The impact of COVID-19 on the RSVs of conspiracy-related queries was evident (Figure 1); in particular, all considered infodemic queries underwent a significant level-shift during the Codogno, Italy, outbreak at the end of February 2020, signaling an immediate increase in fake news with the arrival of the pandemic in Italy (∆_1%_=+102.5, *t*_1_=6.3; ∆_2%_=+288.2, *t*_2_=11.5; Δ*Α*_3%_=+10.2; Δ_4%_=+60.6, *t*_4_=6.2).

It is relevant that the COVID-19 national outbreak has influenced the RSV trend even after the end of the first lockdown (May 2020). Indeed, Figure 1 shows a permanent level-shift for all of the investigated web queries.

Regional interest in conspiracy-related keyword 1 decreased, on average, during the 2016-2020 time-lapse period, from 71 (SEM 4.2) to 52 (SEM 4.1) (Table 1). From 2020 to 2021, the increase in RSV was manifest and common to all regions (
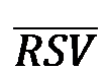
=80 ± 2.8, *t_min_*=1.8, Δ*_min%_*=+12.4; *t_max_*=5.7, Δ*_max%_*=+54.6). Over the 2016-2020 period, the percentage standard deviation ranged from 22.1 to 32.6, while it reached an absolute minimum of 14.4 during the COVID-19 pandemic. This fact shows that web interest in conspiracies has also become more homogeneous across regions. No correlation was sought, as data were subject to a strong dependence on the day of collection [24]; however, the mean values and standard deviations never changed significantly (*t_max_*=0.4).

**Table 1:** Relative search volumes (RSVs) on the web of the keyword “conspiracy + new world order + plot” from 2016 to 2021.

Variable			Value								
			2016-2017		2017-2018		2018-2019		2019-2020		2020-2021
**RSV for each Italian region**											
	Abruzzo	100		76		38		53		67	
	Basilicata	N/A^a^		N/A		N/A		N/A		N/A	
	Calabria	48		61		80		27		78	
	Campania	62		69		59		44		75	
	Emilia-Romagna	74		70		77		68		80	
	Friuli-Venezia Giulia	91		68		39		100		100	
	Lazio	64		85		74		51		84	
	Liguria	81		100		52		69		89	
	Lombardia	85		75		63		43		86	
	Marche	73		68		64		62		91	
	Molise	N/A		N/A		N/A		N/A		N/A	
	Piemonte	56		75		94		58		88	
	Puglia	70		78		45		49		67	
	Sardegna	78		36		100		41		95	
	Sicilia	80		77		62		43		69	
	Toscana	84		75		72		44		80	
	Trentino-Alto Adige	26		41		27		29		54	
	Umbria	70		82		87		49		77	
	Valle d’Aosta	N/A		N/A		N/A		N/A		N/A	
	Veneto	72		54		72		52		84	
**Other statistics**											
	Mean (SD)	71.4 (17.2)		70.0 (15.5)		65.0 (20.3)		51.9 (16.9)		80.2 (11.5)	
	SD%	24.1		22.1		31.2		32.6		14.4	
	SEM (standard error of the mean)	4.2		3.8		4.9		4.1		2.8	
	SEM%	5.9		5.4		7.6		7.9		3.5	
	Shapiro-Wilk *P* value	<.001		.18		.97		.05		.93	

^a^N/A: not applicable due to Google Trends detection anomalies.

On the contrary, web interest in the ByoBlu disinformation channel has always been compatible during the 2016-2021 period (*t* ∈ [–1.3, 1.1], Δ_%_ ∈ [–16.3, 21.8]; *t*_20-21_=–0.1, Δ_20-21_=–1.0; Multimedia Appendix 2).

**Figure 2:**
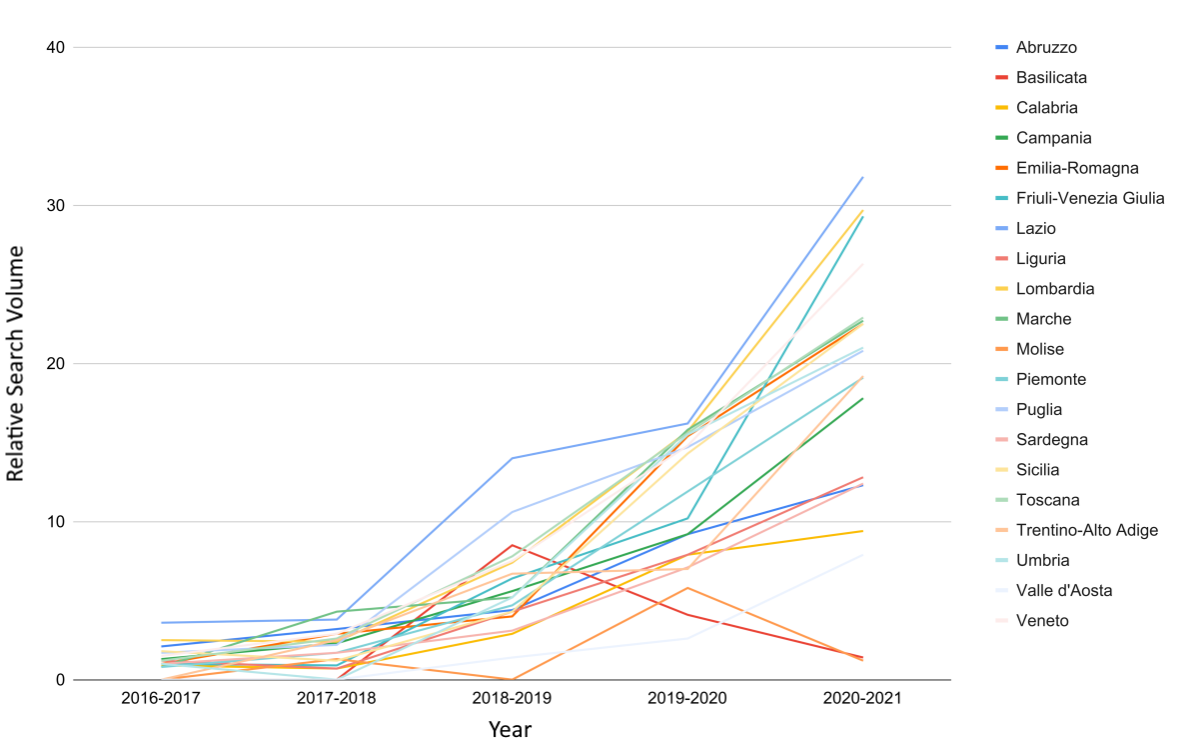
Web interest in the "byoblu" search query by each Italian region from 2016 to 2021.

Figure 1 shows a substantial increase in national searches, and it is evident that the query “byoblu” was searched more in some regions than others. By analyzing the regional trends one by one, it can be observed that web interest in “byoblu” has increased over time, except for in Basilicata and Molise (Figure 2). Although a growing trend was already present, the novel coronavirus seems to have strongly impacted RSVs in Campania (Δ_20-21%_=+93.3 vs Δ_19-20%_=+63.8), Friuli-Venezia Giulia (Δ_20-21%_=+187.6 vs Δ_19-20%_=+59.0), Lazio (Δ_20-21%_=+95.7 vs Δ_19-20%_=+15.8), Trentino-Alto Adige (Δ_20-21%_=+173.2 vs Δ_19-20%_=+4.5, and Valle d’Aosta (Δ_20-21%_=+199.2 vs Δ_19-20%_=+86.2).

### Risk Perception

**Figure 3:**
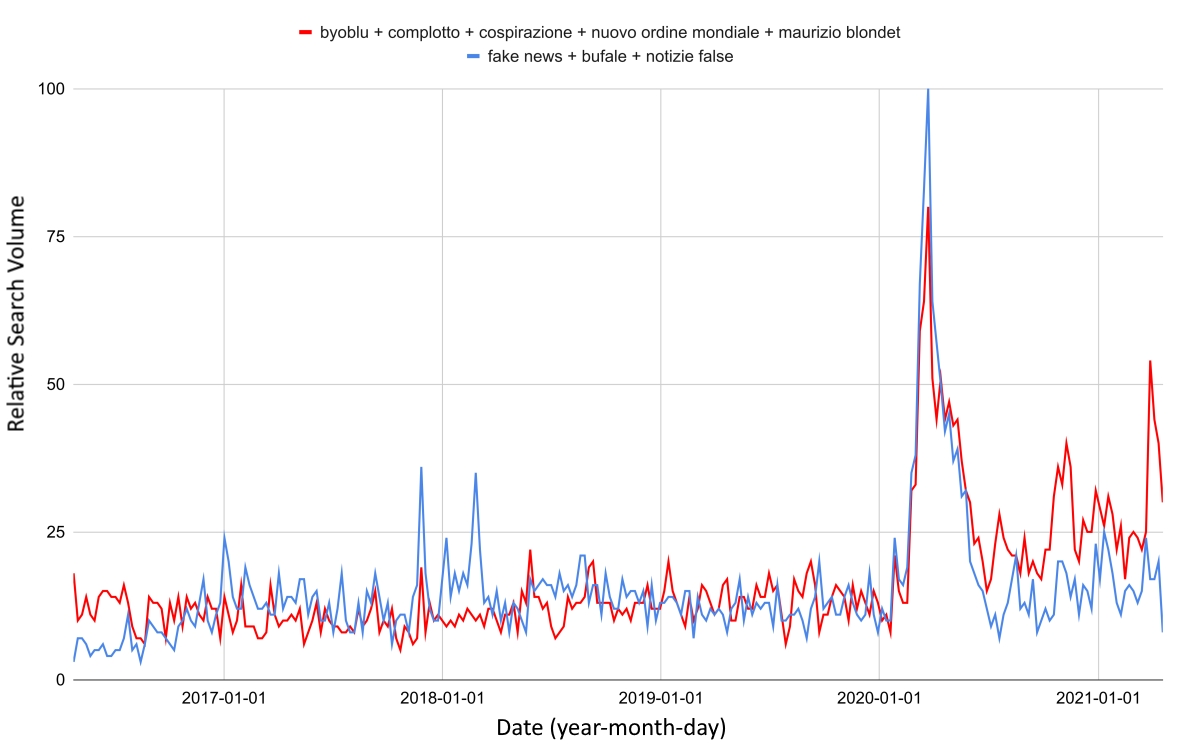
Comparison between web interest in conspiracy hypotheses and antihoax services, by keyword search, from 2016 to 2021.

**Figure 4:**
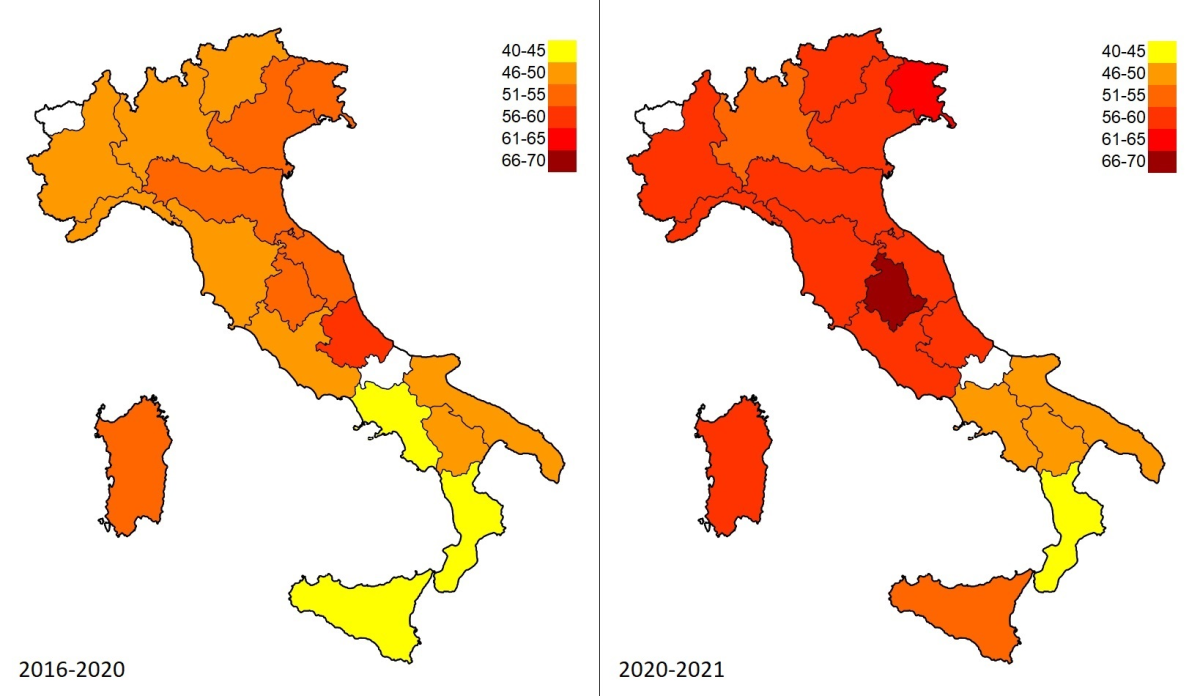
Heat maps comparing web interest in conspiracy hypotheses and antihoax services for each time period in the Italian regions. The index shows the percentage of infodemic queries (eg, 70 means 70% conspiracy-related queries vs 30% antihoax-related queries).

A fraction of the users seemed aware of the danger inherent in COVID-19 fake news circulating on the web and tried to limit its effects by relying on antihoax websites (Figure 3). Nevertheless, the impact of COVID-19 was more incisive for conspiracy hypotheses (*t*_1_=11.3 vs *t*_2_=4.5; Δ_1%_=+157.6 vs Δ_2%_=+84.7), so much so that it is possible to observe a greater level-shift in the trend of infodemic web queries. This worsening was homogeneous on a national scale (Figure 4).

**Figure 5:**
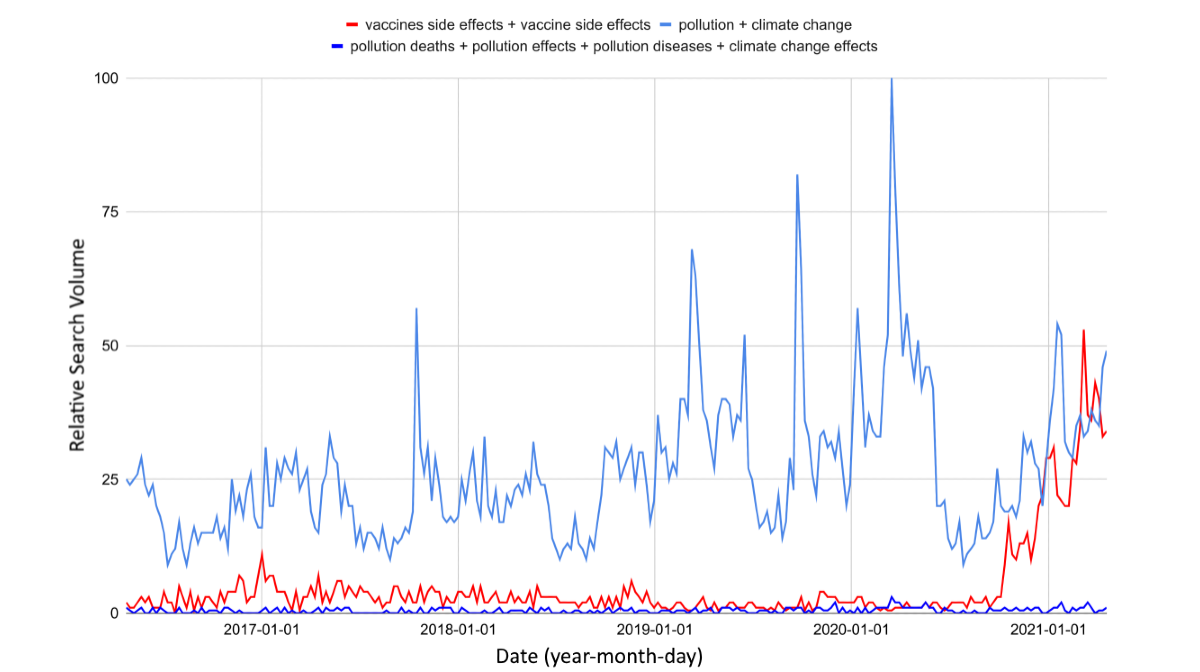
Web interest in vaccine side effects compared with interest in pollution and climate change from 2016 to 2021.

With the announcement of the discovery of COVID-19 vaccines, web interest in side effects immediately soared (*t*_27.1_=10.3, Δ_%_=+905.2 from October 2020 to April 2021). Although health authorities reported rare side effects of adenoviral vector vaccines only, web interest in this topic exceeded interest in pollution and climate change (SS_vaccines_=1.2, *P*<.001 vs SS_pollution_=0.81, *P*<.001; Δ_%_=+44.0; Figure 5), which are much more urgent issues. Queries related to the effects of pollution and climate change returned negligible RSVs.

**Figure 6:**
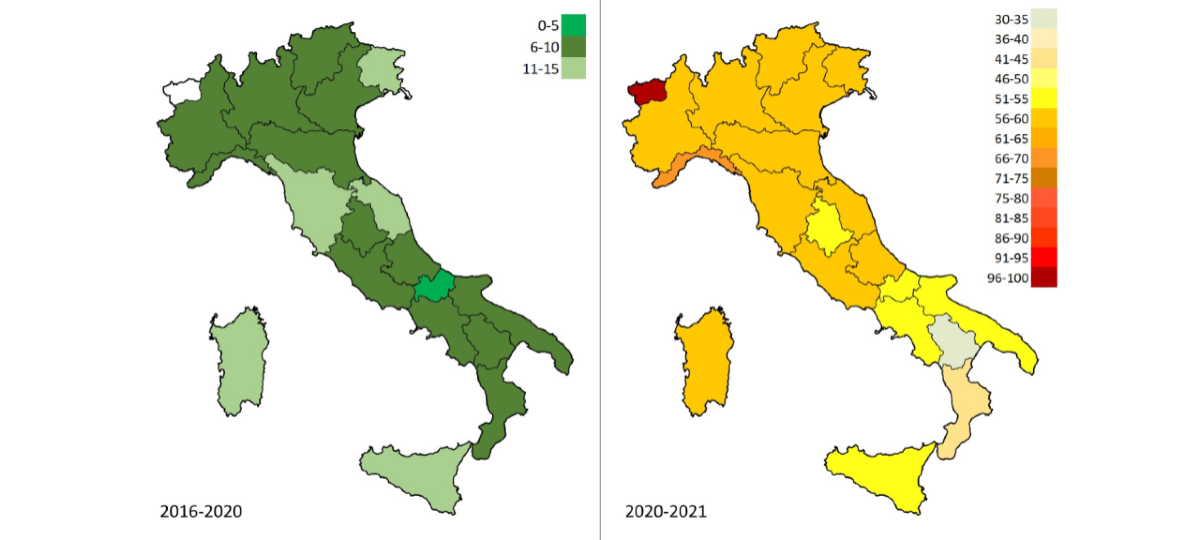
Heat maps comparing web interest in vaccines and web interest in pollution and climate change for each time period in the Italian regions. The index shows the percentage of vaccine-related queries (eg, 70 means 70% queries about vaccine side effects vs 30% queries about pollution and climate change).

When considering queries related to vaccine names, the gap between the RSVs further widened; specifically, the discrepancy between the SS values of the two graphs was substantial, which underlines the unjustified disproportion in risk perception between these two topics (SS_vaccines_=0.22, *P*<.001 vs SS_pollution_=0.05, *P*<.001; Δ_%_=+296.4). As observable in Figure 6, the risk perception in vaccines has surpassed that in pollution and climate change in a homogeneous way throughout Italy.

### COVID-19–Related Fake News

**Figure 7:**
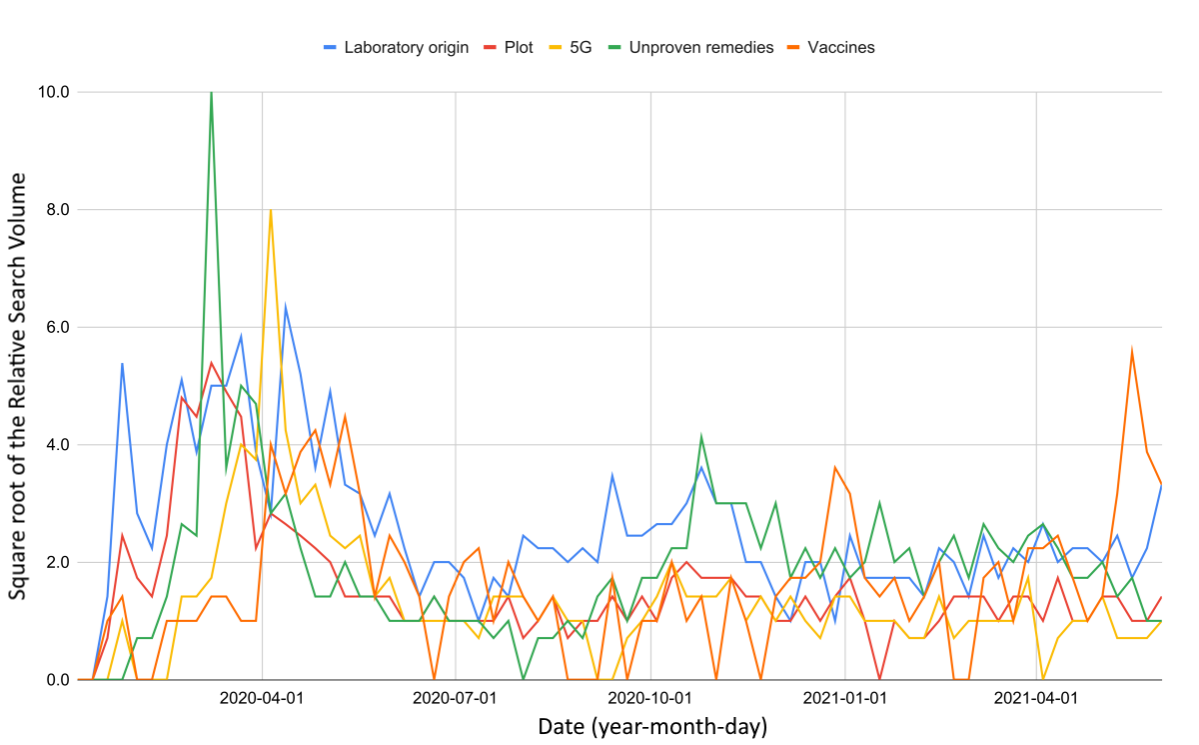
Web interest in COVID-19–related conspiracies over time. The square roots of the relative search volume values have been reported for reasons of readability.

By temporarily excluding vaccines, web interest in COVID-19–related fake news reached its peak during the first wave of the pandemic and then declined, as of April 2021 (Δ_%_ ∈ [–86.1, –73.7], *t* ∈ [–5.7, –2.3]). However, as observable in Figure 7, the trend of keywords related to the engineered novel coronavirus and unproven COVID-19 remedies had stabilized at values significantly far from 0 (
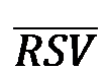
 = 4.8 ± 0.4, 
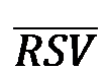
 = 3.8 ± 0.4, respectively). By restricting the domain from January to June 2021—so as to obtain daily RSVs instead of weekly RSVs—a level-shift of web interest in the manufactured origin of SARS-CoV-2 was evident (comparison between May 1 to 22 and May 23 to June 1; Δ_%_=+119.1, *t*_10.3_=2.5). Through the iterative comparison of RSVs, it was possible to estimate that, in the last 12 months, this query represented about 0.04% of COVID-19–related web searches. Regarding vaccines, the highest RSV peak was reached in the week of May 16 to 22, 2021. Such a surge was mainly due to the query “vaccino calamita” (vaccine magnet). By comparing the time-lapse periods of January 1 to May 22, 2021, and May 22 to 31, 2021, a 761% increase in infodemic searches on vaccines was found (*t*_9.5_=6.1).

At the regional level (Table 2), from the beginning of the pandemic, web interest in COVID-19–related fake news was not equally distributed (SD% ∈ [21.4, 33.2]), as opposed to that of generic news (SD%=9.4). Furthermore, Tables 2 and 3 testify to the absence of a regional predisposition for fake news in general and, at the same time, prove a diffused interest in specific topics. Indeed, all of the keywords were low or noncorrelated to each other (|*r*| ∈ [0.01, 0.39], *P* ∈ [.13, .97]).

**Table 2:** Relative search volumes (RSVs) on the web of COVID-19–related fake news from January 2020 to June 2021.

Variable			Value														
			Laboratory origin		Plot		5G		Unproven remedies		Vaccines		General news				
**RSV for each Italian region**																	
	Abruzzo	67		50		57		58		92		86					
	Aosta	N/A^a^		N/A		100		N/A		N/A		84					
	Apulia	100		64		77		53		92		80					
	Basilicata	N/A		74		28		78		N/A		92					
	Calabria	90		100		61		76		100		90					
	Campania	71		62		80		68		46		73					
	Emilia-Romagna	64		69		91		62		64		80					
	Friuli-Venezia Giulia	47		47		91		60		97		87					
	Lazio	84		57		58		68		81		84					
	Liguria	80		42		77		73		45		79					
	Lombardy	63		69		76		98		56		82					
	Marche	72		33		68		92		82		85					
	Molise	N/A		43		N/A		N/A		N/A		85					
	Piedmont	71		78		75		100		71		80					
	Sardinia	85		50		65		72		39		88					
	Sicily	75		69		82		42		50		80					
	Trentino-Alto Adige	44		26		40		55		N/A		66					
	Tuscany	65		53		61		80		89		98					
	Umbria	64		25		71		87		N/A		100					
	Veneto	50		68		67		68		95		76					
**Other statistics**																	
	Mean (SD)	70.1 (15.0)		56.8 (18.9)		69.7 (17.3)		71.7 (15.8)		73.3 (21.5)		83.8 (7.9)					
	SD%	21.4		33.2		24.7		22.0		29.4		9.4					
	SEM (standard error of the mean)	3.6		4.3		4.0		3.7		7.6		1.8					
	Shapiro-Wilk *P* value	.86		.78		.59		.92		.09		.76					

^a^N/A: not applicable due to Google Trends detection anomalies.

**Table 3:** Correlation analysis (relative search volume Pearson *r* and two-tailed *P* value) among topics regarding COVID-19–related fake news in the Italian regions.

Topic			Laboratory origin		Plot		5G		Unproven remedies		Vaccines^a^		General news
**Laboratory origin**													
	*r*	1		0.38		0.06		–0.01		–0.15		0.20	
	*P* value	—^b^		.13		.82		.97		.59		.44	
**Plot**													
	*r*	0.38		1		0.26		0.03		0.15		–0.06	
	*P* value	.13		—		.31		.91		.59		.82	
**5G**													
	*r*	0.06		0.26		1		–0.02		–0.32		0.05	
	*P* value	.82		.31		—		.94		.24		.85	
**Unproven remedies**													
	*r*	–0.01		0.03		–0.02		1		–0.05		0.36	
	*P* value	.97		.91		.94		—		.86		.16	
**Vaccines^a^**													
	*r*	–0.15		0.15		–0.32		–0.05		1		0.39	
	*P* value	.59		.59		.24		.86		—		.15	
**General news**													
	*r*	0.20		–0.06		0.05		0.36		0.39		1	
	*P* value	.44		.82		.85		.16		.15		—	

^a^Only 15 Italian regions were included in this analysis.

^b^Not applicable.

## Discussion

### Principal Findings

To the best of the author’s knowledge, this is the first study to investigate the impact of COVID-19 on pre-existing fake news and the risk perception of Italian web users. These findings show that the pandemic— understood as a set of different situations, such as a health crisis, an economic crisis, lockdowns, disease, and an infodemic—has significantly increased the phenomenon of conspiracies and interest in them. This influence not only caused a marked initial growth of RSV during the first lockdown (March to May 2020) but also generated a pronounced level-shift in web interest that has persisted until at least April 2021. Regional web interest in conspiracy hypotheses during the pre–COVID-19 2016-2020 period had assumed a clear negative trend and was more noticeable in some areas than in others. However, with the advent of the novel coronavirus, interest has increased to reach the highest level in the last 5 years, becoming even more homogeneous across regions. Due to the high dependence of the RSV on the day of gathering, it was not possible to search for correlations with the regional numbers of COVID-19 cases; nevertheless, the mean values and the sample variances have always remained similar. On the contrary, when analyzing the data year by year, no change in average interest in the infodemic YouTube channel ByoBlu, which had over 500,000 subscribers, was observed between the regions. Since a strong increase was highlighted nationwide, some regions must have contributed far more than others to the jump in total RSV. Specifically, as shown in Figure 2, Campania, Lazio, Friuli-Venezia Giulia, Trentino-Alto Adige, and Valle d’Aosta experienced a much higher increase than the other regions. Moreover, a growing trend in RSV during the last 5 years involved all regions except Basilicata and Molise. Finally, web interest in fake news sources has increased more than interest in antihoax services. These results are not to be underestimated; indeed, the Ministry of Health, various online platforms such as YouTube and Twitter, and social networks such as Facebook and Instagram have declared war without borders against the rampant infodemic. Specifically, under each video relating to the pandemic, YouTube has affixed a warning bar that offers users the opportunity to read the latest COVID-19 news on the Ministry of Health official website, complete with a button to access it. A similar procedure has been adopted by Facebook and Instagram. All of these companies have banned accounts and channels that are protagonists of the spread of fake news, including ByoBlu [32-36]. This approach is partly consistent with the procedure proposed by the WHO to deal with the infodemic but was not enough to contain disinformation in Italy. Among the problems that have undermined the effectiveness of these strategies, there is the resonance given by newspapers and television channels to unreliable or misleading information [37-40]. Beyond the mere disinformation contribution, this can foster distrust toward mass media, making the information campaign even more complex during times of crisis [41]. Furthermore, despite all of the countermeasures adopted, social networks and messaging apps, such as WhatsApp, are fake news vehicles [37,38,41].

Alongside this, the influence of newspapers and television news on the risk perception related to COVID-19 vaccines was evident. Although the trend has been on the rise since early October 2020, the headlines of online, printed, and television news publications have often been the subject of criticism from the scientific community as sensationalistic and far from the scientific evidence [42-44]. Distrust of vaccines is a growing issue that raises a serious public health question [45-47]. Although most vaccine-related fake news circulates on social networks, the national mass media must attend to the evidence presented in the scientific literature with appropriate and thoughtful language. In particular, the effect of deliberately misleading titles linked to secondary aspects of the article can have serious consequences [48]. In such an intricate scenario, web interest in vaccine side effects has overtaken interest in pollution and climate change. Notwithstanding that the author of this paper loudly supports the pharmacovigilance process and is aware of the existence of numerous studies on the possible causal link between adenoviral vector COVID-19 vaccines and thrombotic events [49-51], it is necessary to consider that pollution and climate change constitute one of the major global threats today, claiming millions of victims every year [27,52]. Since Janssen and Vaxzevria vaccines have very rare side effects [28,52-55], it is reasonable to conclude that the risk perception of Italian users is distorted and disconnected from the real dangers that menace them. This is even more true when considering the incidence of COVID-19 itself in this type of event [56,57].

Finally, COVID-19 and the crisis it caused have generated fertile ground for new conspiracy hypotheses. While some of these, such as the link between 5G and the spread of the epidemic, have waned over time, others, including the human engineering of the virus in a Wuhan laboratory, phantom infection remedies with no scientific basis, and intentionally altered vaccines, have persisted until today. To further complicate the scenario, the spread of COVID-19–related fake news has not been uniform among the regions; indeed, the RSV groups showed low multicollinearity and vast discrepancies (eg, Abruzzo, Puglia, Calabria, Friuli-Venezia Giulia, and Veneto showed a high interest in the vaccine infodemic and a significantly lower interest in unproven remedies). As a conclusive consideration, the author of this paper emphasizes that it is correct to use the term “infodemic” to describe news that supports any hypotheses without supporting evidence [58]. At the same time, science needs to continue to investigate any possible leads [59].

### Limitations

There are no guarantees that Google Trends is sufficient for investigating the totality of the interests of the Italian public. In particular, internet penetration in Italy is equal to about 74% of the population [60]. Of this fraction, almost 96% use Google as their default online search engine [61]. Therefore, 29% of the Italian population is not considered in this survey. Furthermore, it is not certain that the keywords used in this research included all the terms related to the topics investigated. Indeed, the algorithm with which Google selects the most relevant related queries is unknown (ie, it may not consider web searches pertinent to the discussion). Finally, some relevant keywords may not have been selected for the analysis. Future research could rely on machine learning algorithms for textual analysis to derive the topics of interest to search for on Google Trends.

### Conclusions

COVID-19 has given a significant boost to web interest in conspiracy hypotheses and has made it more uniform across regions. The pandemic accelerated an already-growing trend in users’ interest toward some fake news sources, including the 500,000-subscriber YouTube channel ByoBlu, which was removed from the platform by YouTube for disinformation in March 2021. The risk perception related to COVID-19 vaccines has been so distorted that vaccine side effect–related queries outweighed those relating to pollution and climate change, which are much more urgent issues. Moreover, a large amount of fake news circulated about COVID-19 vaccines, remedies, and origin. Based on these findings, it is recommended that the Italian authorities implement more effective infoveillance systems, and that communication by the mass media be less sensationalistic and more consistent with the available scientific evidence. In this context, Google Trends can be used to monitor the users’ response to specific infodemiological countermeasures. Further research is needed to understand the psychological mechanisms that regulate risk perception.

## Appendix

Multimedia Appendix 1 Analysis of the degree of an infodemic using keywords mined from Bufale.net.

Multimedia Appendix 2 Details of the analysis.

## Abbreviations

ADF: augmented Dickey-Fuller
HCoV-EMC/2012: human coronavirus–Erasmus Medical Center/2012
MK: Mann-Kendall
RSV: relative search volume
SEM: standard error of the mean
SS: Sen slope
WHO: World Health Organization
