# Supplementary material for: The Impact of COVID-19 on Conspiracy Hypotheses and Risk Perception in Italy: Infodemiological Survey Study Using Google Trends
Source: JMIR Infodemiology. 2021 Aug 6;1(1):e29929. doi: 10.2196/29929 (PMC8363126; doi:10.2196/29929)
Supplement: Multimedia Appendix 1 [file infodemiology_v1i1e29929_app1.docx]

**Multimedia Appendix 1**

Analysis of the degree of infodemic of the keywords mined from Bufale.net. In particular, Bufale.net is an anti-hoaxes site also exploited by other infodemiological papers [1, 2].

1) ByoBlu: this channel - in addition to being removed from YouTube for disinformation - is known in the scientific literature for sharing fake news and conspiracy hypotheses in Italy [1, 2].

2) Maurizio Blondet: infodemic news source such as *“Coronavirus: Pfizer vaccine killed 40 times older than Covid”* (Supplementary Figure 1) [3]. No peer-reviewed scientific references have been cited to justify this claim. Furthermore, Blondet points out that the vaccine was developed to kill the elderly with the phrase “it is its purpose.”


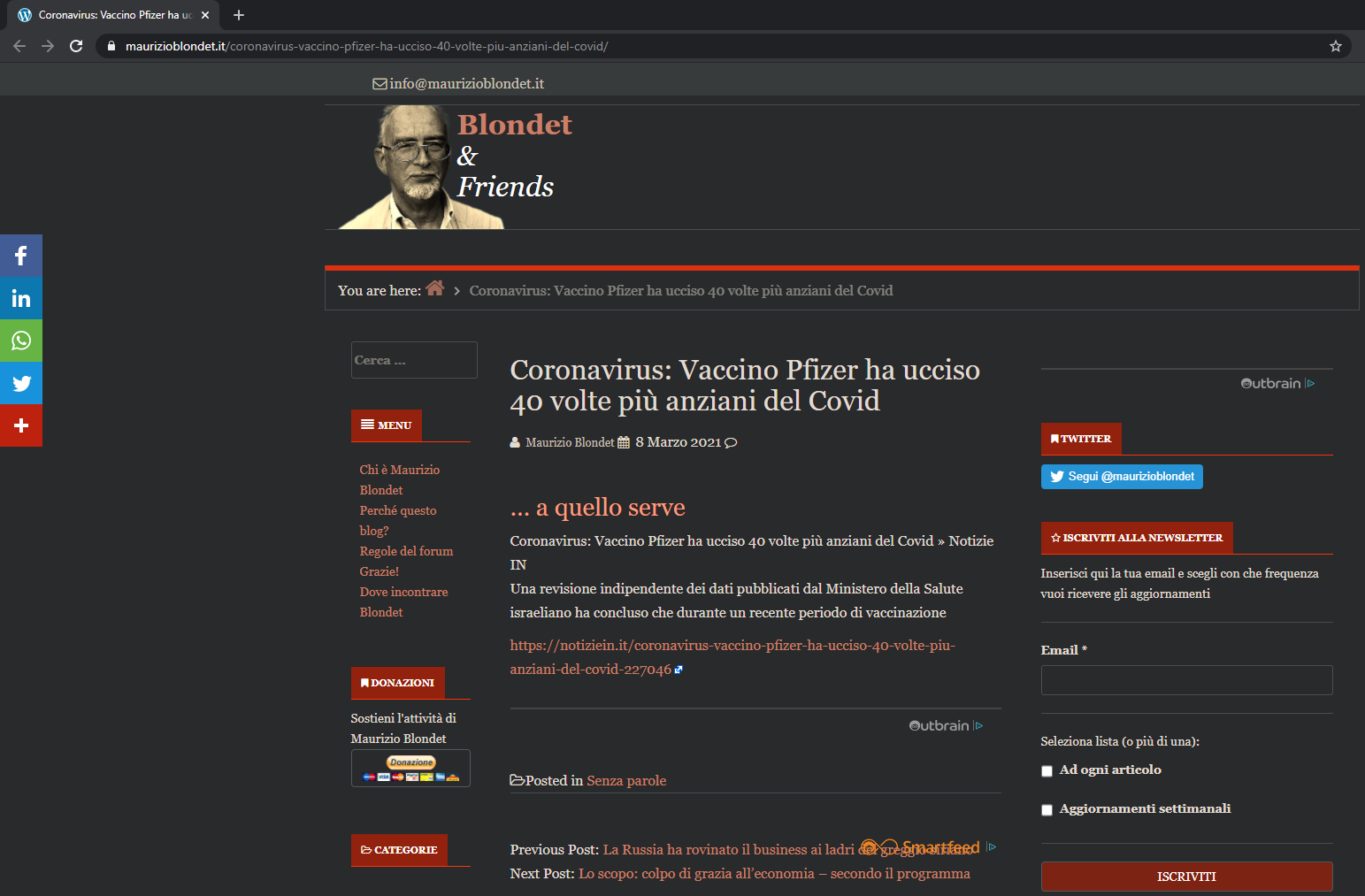
 **Figure S1.** Screenshot of the fake news “Coronavirus: Pfizer vaccine killed 40 times older than Covid” shared by Maurizio Blondet.

3) Luogocomune: infodemic news source such as *“The coronavirus conspiracy”* (Supplementary Figure 2) [4].


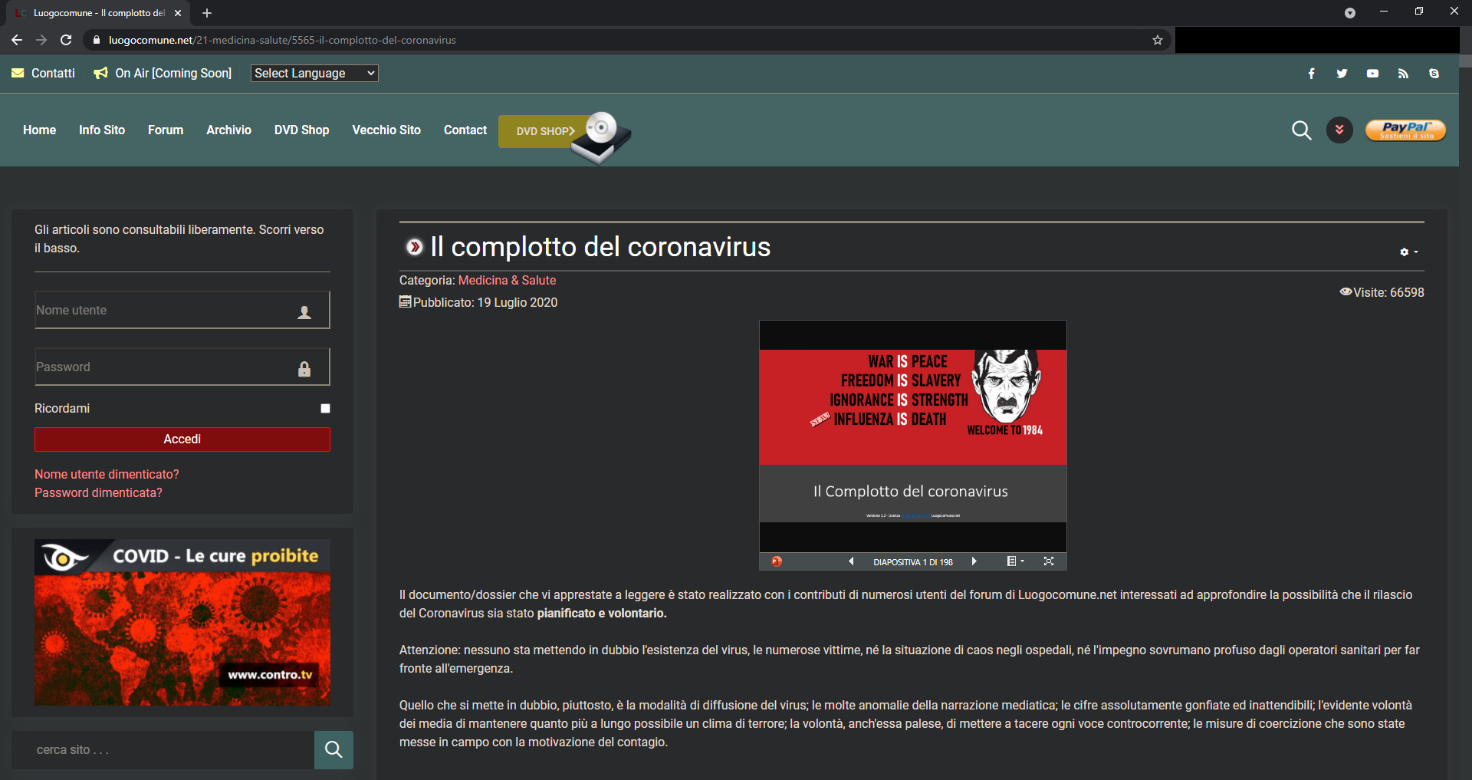
 **Figure S2.** Screenshot of the fake news “The coronavirus conspiracy” shared by “Lugocomune.net”

**References**

[1] Pierri F, Artoni A, Ceri S. Investigating Italian disinformation spreading on Twitter in the context of 2019 European elections. PLoS One. 2020 Jan 17;15(1):e0227821. doi: 10.1371/journal.pone.0227821. PMID: 31951628; PMCID: PMC6968875. URL: <https://www.ncbi.nlm.nih.gov/pmc/articles/PMC6968875/>

[2] Pierri F, Artoni A, Ceri S. HoaxItaly: a collection of Italian disinformation and fact-checking stories shared on Twitter in 2019. arXiv:2001.10926v1 [Preprint]. 2020, Jan 29. URL: <https://arxiv.org/abs/2001.10926>

[3] Blondet M. Coronavirus: Vaccino Pfizer ha ucciso 40 volte più anziani del Covid. [Accessed: 2021, Apr 2] URL: <https://www.maurizioblondet.it/coronavirus-vaccino-pfizer-ha-ucciso-40-volte-piu-anziani-del-covid/>

[4] Luogocomune.net. Il complotto del coronavirus. 2020, Jul 19. [Accessed: 2021, Apr 2]. URL: <https://www.luogocomune.net/21-medicina-salute/5565-il-complotto-del-coronavirus>
